# Proliferating CD8^+^ T Cell Infiltrates Are Associated with Improved Survival in Glioblastoma

**DOI:** 10.3390/cells10123378

**Published:** 2021-12-01

**Authors:** Ileana S. Mauldin, Jasmin Jo, Nolan A. Wages, Lalanthica V. Yogendran, Adela Mahmutovic, Samuel J. Young, Maria Beatriz Lopes, Craig L. Slingluff, Loren D. Erickson, Camilo E. Fadul

**Affiliations:** 1 Department of Surgery, University of Virginia, Charlottesville, VA 22903, USA; sjy2ac@hscmail.mcc.virginia.edu (S.J.Y.); CLS8H@hscmail.mcc.virginia.edu (C.L.S.J.); 2 Cancer Center, University of Virginia, Charlottesville, VA 22903, USA; am6bd@virginia.edu; 3 Department of Internal Medicine, Division of Hematology and Oncology, East Carolina University, Greenville, NC 27834, USA; Jasmin.Jo@Vidanthealth.com; 4 Department of Public Health Sciences, Division of Translational Research and Applied Statistics, University of Virginia, Charlottesville, VA 22904, USA; naw7n@virginia.edu; 5 Department of Neurology, Division of Neuro-Oncology, University of Virginia, Charlottesville, VA 22903, USA; lvy3sy@hscmail.mcc.virginia.edu (L.V.Y.); cef3w@hscmail.mcc.virginia.edu (C.E.F.); 6 Department of Pathology, Division of Neuropathology, University of Virginia Health System, Charlottesville, VA 22908, USA; MSL2E@hscmail.mcc.virginia.edu; 7 Department of Microbiology, Immunology, and Cancer Biology, University of Virginia School of Medicine, Charlottesville, VA 22908, USA; lde9w@eservices.virginia.edu; 8 Beirne B. Carter Center for Immunology Research, University of Virginia School of Medicine, Charlottesville, VA 22908, USA

**Keywords:** immunology, tumor infiltrating lymphocytes, multiplex immunofluorescence histology, glioblastoma, human

## Abstract

Background: tumor-infiltrating lymphocytes are prognostic in many human cancers. However, the prognostic value of lymphocytes infiltrating glioblastoma (GBM), and roles in tumor control or progression are unclear. We hypothesized that B and T cell density, and markers of their activity, proliferation, differentiation, or function, would have favorable prognostic significance for patients with GBM. Methods: initial resection specimens from 77 patients with IDH1/2 wild type GBM who received standard-of-care treatment were evaluated with multiplex immunofluorescence histology (mIFH), for the distribution, density, differentiation, and proliferation of T cells and B cells, as well as for the presence of tertiary lymphoid structures (TLS), and IFNγ expression. Immune infiltrates were evaluated for associations with overall survival (OS) by univariate and multivariate Cox proportional hazards modeling. Results: in univariate analyses, improved OS was associated with high densities of proliferating (Ki67^+^) CD8^+^ cells (HR 0.36, *p* = 0.001) and CD20^+^ cells (HR 0.51, *p* = 0.008), as well as CD8^+^Tbet^+^ cells (HR 0.46, *p* = 0.004), and RORγt^+^ cells (HR 0.56, *p* = 0.04). Conversely, IFNγ intensity was associated with diminished OS (HR 0.59, *p* = 0.036). In multivariable analyses, adjusting for clinical variables, including age, resection extent, Karnofsky Performance Status (KPS), and MGMT methylation status, improved OS was associated with high densities of proliferating (Ki67^+^) CD8^+^ cells (HR 0.15, *p* < 0.001), and higher ratios of CD8^+^ cells to CD4^+^ cells (HR 0.31, *p* = 0.005). Diminished OS was associated with increases in patient age (HR 1.21, *p* = 0.005) and higher mean intensities of IFNγ (HR 2.13, *p* = 0.027). Conclusions: intratumoral densities of proliferating CD8 T cells and higher CD8/CD4 ratios are independent predictors of OS in patients with GBM. Paradoxically, higher mean intensities of IFNγ in the tumors were associated with shorter OS. These findings suggest that survival may be enhanced by increasing proliferation of tumor-reactive CD8^+^ T cells and that approaches may be needed to promote CD8^+^ T cell dominance in GBM, and to interfere with the immunoregulatory effects of IFNγ in the tumor microenvironment.

## 1. Introduction

In spite of aggressive treatment with surgery, radiation, and chemotherapy, patients diagnosed with glioblastoma (GBM) have a median survival of about 15 months, and less than 10% are alive 5 years after diagnosis [1]. Relevant prognostic variables for GBM include age, Karnofsky Performance Status (KPS), and methylation status of the *O6-methylguanine-DNA-methyltransferase* (MGMT) gene promoter [2]. The presence and composition of immune cell infiltrates are prognostic in many cancers [3,4,5]. Tumor infiltrating lymphocytes (TIL) have prognostic value in a variety of cancers [3,4,5,6,7]; with a survival advantage associated with the presence of tumor infiltrating CD3^+^ T cells and cytotoxic CD8^+^ T cells [5]. However, studies examining the association of lymphocyte infiltration and prognosis in GBM have rendered contradictory findings; with some studies supporting an association between T cell density and survival and others not [8,9,10,11,12,13,14]. The differing results may be explained in part by the use of different methods of isolation and measurement of the immune cells, the inclusion of lower grade gliomas in the analyses, and the lack of studies evaluating markers of function of TIL [15].

Activated TIL are known to be critical in controlling tumor progression [16,17,18,19]. Markers of T cell activation [7,20], proliferation [21], differentiation, and immune function provide more information about the activity of TIL infiltrating cancer tissue than enumeration alone. The proliferative activity of CD8^+^ T cells, assessed by Ki67 expression, has been reported as a favorable prognostic factor for human renal cell carcinoma [21]. The Th1 T cell differentiation phenotype, mediated by the expression of transcription factor T-bet (TBX21), is associated with favorable outcomes in patients with other cancers [7,20]. Activation can lead to the production of effector cytokines, including IFNγ from Th1 lineage T cells and cytotoxic T cells [22]. Th17 cells expressing the transcription factor retinoic acid receptor-related orphan receptor gamma t (RORγt^+^) have been associated with cancer progression and eradication [23,24,25,26]. Th17 cells expressing IFNγ can be tumor-cytolytic, while those expressing immune suppressive cytokine IL-10, promote tumor growth. Additionally, the cytokine IL-17(A/F), which is produced by Th17 cells, may also promote tumor growth, via acting on tumor cells or other immune cells [26]. In GBM, however, studies of these markers have been limited and data on the prognostic significance of TIL distribution within the tumors have been inconsistent [11,27].

Additionally, B cell infiltrates and their function in GBM have been largely unstudied. B cells have both positive and negative roles in tumor immunity, highlighting the heterogeneity of B cell responses [16,28]. B cells can produce anti-tumor antibodies, suppress antitumor immune responses, as Bregs, or can act as antigen presenting cells (APC) for T cells, thus helping to prime immune responses at the tumor site [28,29,30]. Tertiary lymphoid structures (TLS), which harbor dense B cell infiltrates, are ectopic lymphoid organs that develop in non-lymphoid tissues, and have been associated with improved survival in several cancers [31,32,33,34,35,36,37,38,39].

To advance the understanding of the prognostic value of TIL in GBM, we evaluated the distribution and density, of T cell and B cells in GBM, and their expression of markers associated with immune activity, by multiplex immunofluorescence histology (mIFH). We hypothesized that GBM specimens containing increased densities of proliferating T or B cells or Th1 lineage cells, increased IFNγ expression, or TLS, would have favorable prognostic significance for patients with GBM.

## 2. Materials and Methods

### 2.1. Patients and Database

The University of Virginia Neuro-Oncology tumor bank (IRB Protocol# 13163), which currently houses GBM specimens from 197 unique cases, was the source of tissue for these studies. Available specimens and their clinical data were reviewed for eligibility. Selection criteria included only cases with formalin-fixed paraffin-embedded (FFPE) tissue, cases that were diagnosed at least 2 years prior to the study start, and patients who received standard of care treatment for GBM, defined as maximum feasible surgical resection followed by concomitant radiation therapy and temozolomide (TMZ). Of 197 unique cases, 108 specimens had evaluable tissue available, of which 77 patients/specimens met selection criteria and were included in this study. Reasons for exclusion included: specimens were from a second operation (*n* = 6), review of pathology revealed anaplastic oligodendroglioma and not GBM (*n* = 1), patients received radiation therapy only, or treatment status unknown (*n* = 4), patients received only supportive treatment after surgery (*n* = 8), patients did not complete RT and TMZ (*n* = 7), or GBM had IDH1/2 mutation (*n* = 5). The data for survival and recognized prognostic factors were obtained from the Neuro-Oncology database (IRB protocol #11740). The extent of resection was determined by brain MRI performed within 72 h after surgery, except for patients undergoing only biopsy. Intraoperative post-resection MRI was also acceptable. KPS score was obtained from the electronic medical record. When not documented, a KPS score was calculated from the physician notes [26]. Use of human tissues was approved by the UVA Institutional Review Board (IRB protocol no. 20210). A neuropathologist (MBL) verified the surgical pathology material according to the 2016 WHO classification guidelines, and helped in identifying representative tumor areas on hematoxylin and eosin (H&E) stained slides from each block.

### 2.2. Immunohistochemistry and Image Analysis

In total, four micrometre thick sections were cut from formalin fixed paraffin embedded (FFPE) tissue specimens, and human lymph node and bowel were used as positive control tissues. Multispectral staining was performed according to the manufacturer’s protocol using the OPAL Multiplex Manual IHC kit, and antigen retrieval buffers (AR) 6 and 9 (Akoya Biosciences, Marlborough, MA, USA). Staining sequence, antibodies, and antigen retrieval buffers were performed on three serial sections as follows:

TIL Panel 1: AR9, CD4 (1:100, clone SP35) (Cell Marque, Rocklin, CA, USA) Opal520; AR9, CD8 (1:500, clone C8/144B) (Agilent, Santa Clara, CA, USA) Opal540; AR6, CD20 (1:4k, clone L26) (Agilent, Santa Clara, CA, USA) Opal570; DIVA, CD34 (1:200, clone QBEnd 10) (Agilent, Santa Clara, CA, USA) Opal620; DIVA, T-bet (1:50, clone 4B10) (Santa Cruz Biotechnology, Dallas, TX, USA) Opal650; and spectral DAPI (Akoya Biosciences, Marlborough, MA, USA).

TIL Panel 2: AR9, CD4 (1:100, clone SP35) (Cell Marque, Rocklin, CA, USA) Opal520; AR9, CD8 (1:500, clone C8/144B) (Agilent, Santa Clara, CA, USA) Opal540; AR6, CD20 (1:4k, clone L26) (Agilent, Santa Clara, CA, USA) Opal570; DIVA, IFNγ (1:1k, clone IFNG/466) (NeoBiotechnologies, Union City, CA, USA) Opal620; and Ki67 (1:20, clone SP6) (Abcam, Waltham, MA, USA) and spectral DAPI (Akoya Biosciences, Marlborough, MA, USA).

TLS Panel 3: DIVA, MAdCAM (1:200, clone355G8) (Invitrogen, Carlsbad, CA, USA) Opal620; AR9, CD8 (1:500, clone C8/144B) (Agilent, Santa Clara, CA, USA) Opal540; AR6, CD20 (1:1k, clone L26) (Agilent, Santa Clara, CA, USA) Opal570; AR6, PNAd (1:100, clone MECA-79) (BD Pharmingen, Franklin Lakes, NJ, USA) Opal650; DIVA, RORγt (1:1k, clone 6F3.1) (EMD Millipore, Burlington, MA, USA) Opal570; and spectral DAPI (Akoya Biosciences, Marlborough, MA, USA). Images of slides stained with these 3 mIFH panels are shown in Supplemental Figures S1–S3, as well as staining controls.

OPAL Polymer HRP Ms + Rb (cat# ARH1001EA) (Akoya Biosciences, Marlborough, MA, USA) was utilized for all stains except for the PNAd stain which utilized Super Picture HRP Polymer Conjugate Broad Spectrum antibody (cat# 87-8963, Life Technologies, Carlsbad, CA, USA).

Stained slides were mounted using prolong diamond antifade (Life Technologies, Carlsbad, CA, USA) and scanned at 10× magnification using the PerkinElmer Vectra 3.0 system and Vectra software (Akoya Biosciences). Regions of interest were selected in Phenochart software, and 20× magnification images were acquired with the Vectra 3.0 system. Multiplex stain samples were compared to H&E-stained serial sections to guide viable tissue selection (Supplemental Figure S4). These images were spectrally unmixed using single stain positive control images in the InForm software (Akoya Biosciences, Marlborough, MA, USA). Cell infiltrates were enumerated using HALO software (Indica Labs, Albuquerque, NM, USA). Cell counts were normalized to per mm^2^ of tissue. The ratio of CD8 to CD4 cells was calculated by dividing the number of CD8^+^ cells per mm^2^ tissue by CD4^+^ cells per mm^2^ tissue. Immunotype A, B, and C patterns of immune cell infiltration, previously used in melanoma, were used to define immune infiltrate patterns in GBM tumors [27]. GBM that contained low (≤50 cells/mm^2^) B and T cell infiltrates (CD4^+^ and CD8^+^ cells) were defined as immunotype A, immunotype B tumors contained B and T cell infiltrates (>50 cells/mm^2^) localized near blood vessels, and immunotype C tumors contained B and T cell infiltrates (>50 cells/mm^2^) localized diffusely throughout tumor.

### 2.3. Statistical Analysis

We followed REMARK guidelines in the design and reporting of the study (Supplemental Table S1) [40]. For each tissue section, cell counts were enumerated per mm^2^. CD8^+^ and CD20^+^ cells were stained in all 3 mIFH panels, which provided 3 serial FFPE sections to enumerate those cells; CD4^+^ cells were stained and enumerated from 2 mIFH panels, those values were averaged. Mean analyzed tissue areas are shown in Supplemental Figure S5A, and count variances among the analyzed slides are shown in Supplemental Figure S5B. Spearman’s rank correlation coefficient was used to assess associations among continuous variables between the mIFH panels. We observed significant (*p* < 0.0001 for all) and positive correlations between CD4, CD8, and CD20 markers across the panels and serial sections (Supplemental Figure S5B). Other immune markers were assessed from a single FFPE section. The main endpoint for analysis was overall survival, defined as the time from initial surgery to death. Patients who were alive at last follow-up were censored. The Kaplan–Meier product limit estimator [41] was used to estimate survival distributions. Univariate and multivariate Cox proportional hazards modeling was performed using R software version 3.5.3. Cox proportional hazards assumptions were tested using Schoenfeld residual analysis for univariate and multivariate analysis using a level of significance of 0.01. For all analyzed immune markers, densities were dichotomized into “high” and “low” classifications, using the method of Contal and O’Quigley [42] to determine the optimal cutoff for continuous covariates with time-to-event outcomes. A stepwise elimination procedure was used for determining the final Cox multiple regression model. Multivariate Cox proportional hazards models assessed the relationship between survival and immune cell densities (dichotomized into “high” and “low” classifications) in conjunction with a set of clinical covariates that included age, KPS score (high > 70 vs. low ≤ 70), extent of initial resection (gross tumor resection (GTR) vs. subtotal resection [STR] vs. Biopsy only), and MGMT promoter methylation. Associations were considered significant for two-sided *p* values ≤ 0.05.

## 3. Results

### 3.1. Patient Demographics

Of 197 cases, 82 GBM specimens from 82 patients that met selection criteria were evaluated for immune infiltrates. We excluded five patients with mutations in isocitrate dehydrogenase 1/2 (IDH) because these patients have improved prognosis compared to patients with wild type IDH [43,44,45]. The median OS in patients with IDH1/2 mutated GBM was 48.3 months, and 17.1 months in patients containing wild type GBM (Supplemental Table S2). Additional details on clinical variables evaluated in patients containing IDH wild type and mutant GBM are shown in Supplemental Table S2. Thus, our final cohort was comprised of 77 patients (45 males, 32 females) with IDH wild type GBM. The mean age at surgery was 61 years: 35 cases (45.5%) had subtotal resections (STR), 35 (45.5%) had gross total resections (GTR), and 7 (9%) had biopsies. Among the 77 samples, 29 (37.7%) contained MGMT gene promoter methylation, 32 (41.6%) were unmethylated, and the methylation status of 16 (20.7%) samples was unknown. KPS score was ≤70 in 41 (53%) patients. The mean duration of perioperative steroid use was 4.8 days; the number of cycles of adjuvant TMZ was 3.8, and the number of cycles of bevacizumab (Bev) was 5.0. Additional details on these clinical variables in patients containing IDH wild type GBM are provided in Table 1.

### 3.2. Distribution and Enumeration of Tumor Infiltrating Lymphocytes

**Figure 1:**
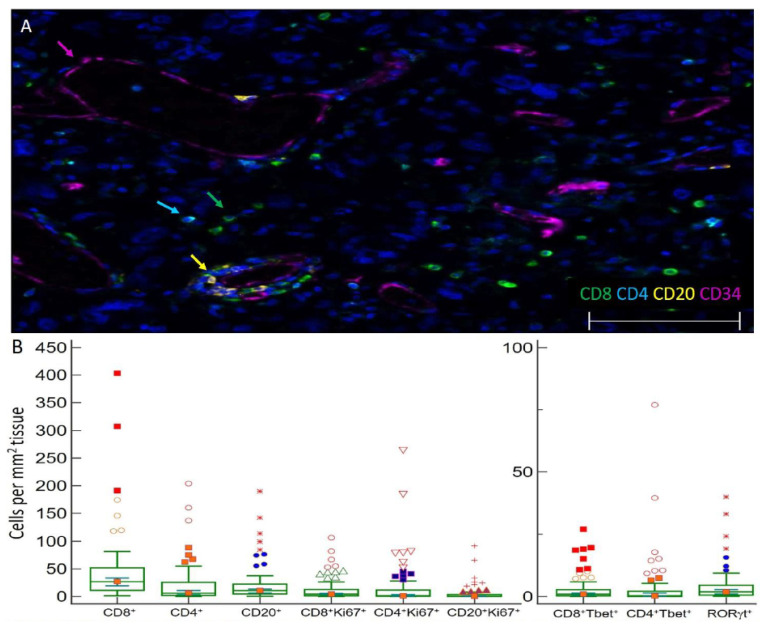

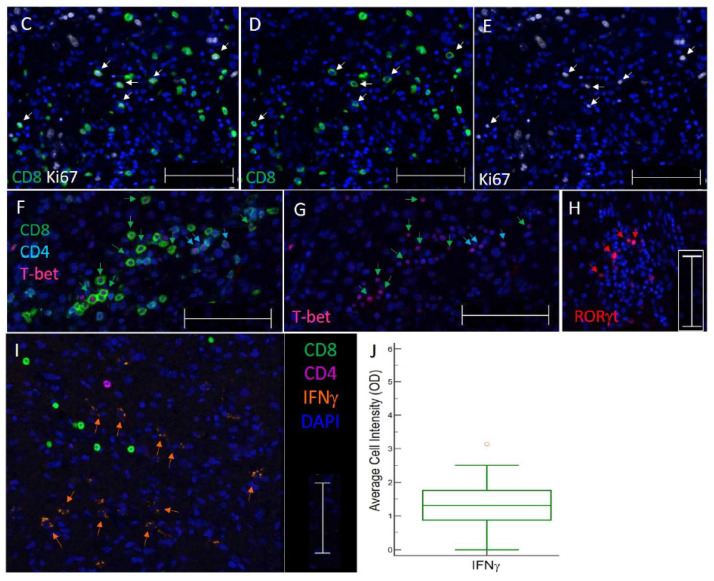
Images of TIL in GBM and immune cell densities. (**A**) Representative 5 color mIFH image of T (CD4^+^ and CD8^+^) and B cells (CD20^+^) localized near CD34^+^ endothelial cells in GBM. Colored arrows denote one example of each cell type in image. (**B**) Box plots of the densities of immune cell subsets per mm^2^ tissue within GBM. The central box represents values from the lower to upper quartile, 25th to 75th percentile. Middle bar identifies median, and whiskers show minimum and maximum, outliers are displayed as separate points. (**C**–**E**) Images of proliferating CD8^+^ T cells in GBM. Image C is a 3 color mIFH image showing CD8^+^ T cells and proliferating Ki67^+^ cells, Image D depicts CD8^+^ cells, and image E is of Ki67^+^ cells, white arrows in each image point to proliferating CD8^+^Ki67^+^ cells. (**F**,**G**) Image of T-bet^+^ T cells in GBM. Image F is a 4 color mIFH image depicting CD8^+^ or CD4^+^ T cells and T-bet nuclear staining, image G shows T-bet expression alone, green arrows denote CD8^+^T-bet^+^ cells and cyan arrows denote CD4^+^T-bet^+^ cells. (**H**) Image of RORγt^+^ cells in GBM, nuclear staining of RORγt^+^ is shown in red, and is denoted by red arrows. (**I**) Representative Image of IFNγt^+^ cells in GBM. Image I is a 4 color mIFH image depicting CD4^+^ or CD8^+^ T cells and IFNγ expression in GBM. For all images colors are indicated, scale bars are 100 μm and DAPI nuclear staining is shown in Blue. (**J**) Box plot of the average cell intensities of IFNγin GBM.

Initial resection specimens were evaluated for infiltrating lymphocytes (CD20^+^ B cells and CD4^+^ or CD8^+^ T cells by mIFH (Figure 1A). The mean area of tumor assayed for infiltrates per FFPE section was 45 mm^2^. T cells expressing CD4 or CD8, CD20^+^ B cells, and subsets of these cells expressing markers of immune activity were enumerated and reported per mm^2^ of tissue (Figure 1B). Median numbers of CD8, CD4, and CD20 infiltrates were 26.9, 6.0, and 10.6 cells/mm^2^ of tissue, respectively (Figure 1B). CD4^+^, CD8^+^, and CD20^+^ TIL densities were also evaluated in five patients with IDH1/2 mutated GBM and were comparable to IDH wild type GBM specimens (Supplemental Table S2). GBM were also evaluated for immunotype. Immunotype A, B, and C patterns of immune cell infiltration, previously used in melanoma, define tumors that have low immune infiltrates (≤50 cells/mm^2^), perivascular infiltrates, or diffuse infiltrates, respectively [46]. Of the 77 IDH wild type tumors, 41 (53%) were immunotype A harboring low T and B cell infiltrates (≤50 cells/mm^2^) and 36 (47%) were immunotype B containing T and B cell infiltrates localized near blood vessels: no immunotype C specimens were evident with diffuse cell infiltrates. Associations between CD4^+^, CD8^+^, and CD20^+^ TIL densities and dexamethasone dose pretreatment, duration, and total dose (dichotomized into high and low) were assessed using a two-sample *t*-test. We did not observe significant associations between dexamethasone treatment and CD4^+^, CD8^+^, or CD20^+^ TIL densities (Supplemental Table S3).

### 3.3. TIL Subsets Express Markers of Cell Proliferation and Differentiation in GBM

We hypothesized that markers of B and T cell proliferation, differentiation, or activity would be associated with prognosis. To evaluate the proliferative status of T and B cells in GBM, we probed for Ki67. Ki67 identified proliferating CD8^+^ T cells (median 4.3/mm^2^, 14.7% of CD8^+^ cells, Figure 1B–E), CD4^+^ T cells (median 1.3/mm^2^, 31.1% of CD4^+^ cells), and B cells (median 0.3/mm^2^, 8.1% of CD20^+^ cells) (Figure 1B) as well as proliferating tumor cells. To evaluate the differentiation status of lymphocytes infiltrating GBM, we probed for transcription factors T-bet (Figure 1F,G) and RORγt (Figure 1H) to enumerate Th1 and Th17 lineage cells, respectively. GBM contained CD4^+^T-bet^+^ cells (median 0.3 cells/mm^2^, representing 4.2% of total CD4, Figure 1B,F,G) and CD8^+^Tbet^+^ cells (median 0.9 cells/mm^2^, representing 3.9% of total CD8 cells, Figure 1B,F,G), as well as RORγt^+^ cells (median of 1.9 cells/mm^2^, Figure 1B,H). IFNγ was probed to investigate effector activities of infiltrating CD4^+^ or CD8^+^ T cells; however, IFNγ was not primarily localized adjacent to the T cell membrane (Figure 1I). GBM contained a median of 1.6 IFNγt^+^ cells/mm^2^. The mean cell intensity of IFNγ in GBM tissues was also evaluated, which ranged in optical density from 0–3.1 with a median of 1.3 (Figure 1J).

### 3.4. Some B and T Cell Aggregates Resemble Immature TLS in GBM

**Figure 2:**
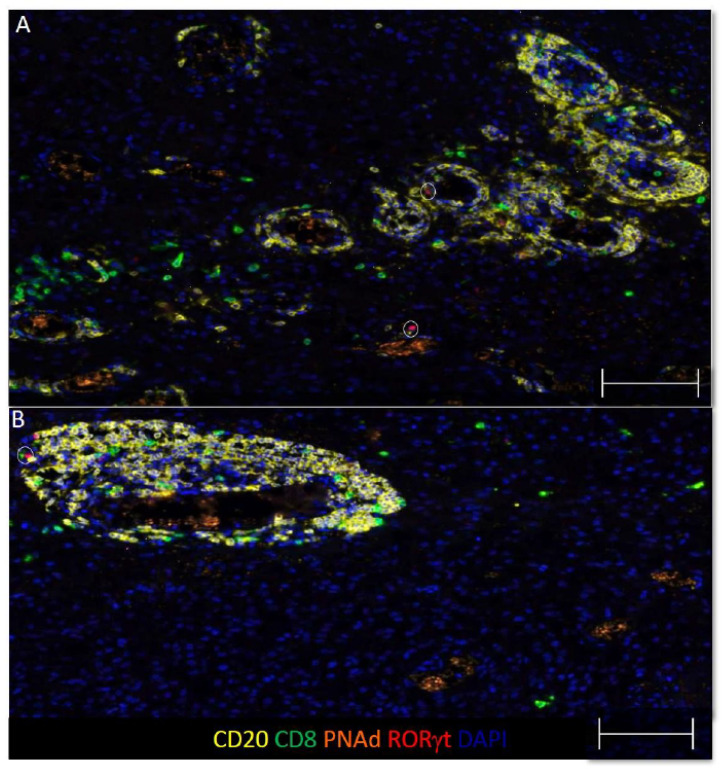
GBM contain focal B and T cell aggregates resembling immature TLS. (**A**,**B**) Representative images of dense clusters of CD20^+^ B cells, CD8^+^ T cells, and PNAd^+^ vasculature from two GBM specimens. The circles denote RORγt^+^ cells, scale bars are 100 μm and colors are indicated.

TLS structures have been found in a number of human solid tumors, where they are associated with improved OS. Since GBM contain B cell infiltrates, we hypothesized that some of these focal B cell infiltrates would be TLS. TLS structures typically contain B and T cells localized near high endothelial venules (HEV), which expresses peripheral lymph node addressin (PNAd) or mucosal vascular addressin cell adhesion molecule 1 (MAdCAM-1). We evaluated focal B and T cell aggregates in GBM for associations with vasculature expressing PNAd or MAdCAM-1. We found that 6 out of 77 (8%) tumors contained aggregates of B and T cells localized near PNAd^+^ vasculature, (Figure 2A,B and Supplemental Figure S3) MAdCAM-1 expression was not observed. As no clear germinal center is evident, these structures resemble immature TLS in appearance and composition [47,48,49].

### 3.5. Subsets of TIL and Markers of Immune Activity Are Associated with Survival in GBM Patients

**Figure 3:**
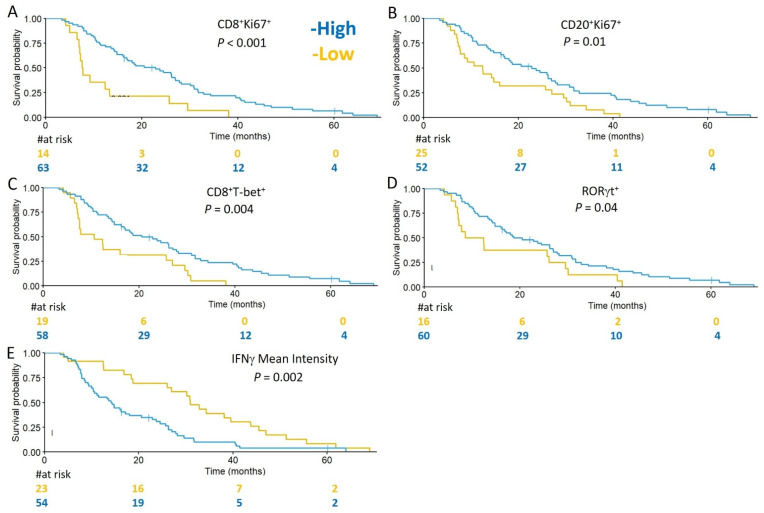
Markers of immune activity are associated with OS in GBM. Kaplan–Meier curves for the indicated immune markers, dichotomized into high (blue) and low (yellow), are shown in (**A**–**E**). Prolonged OS is associated with tumors containing high densities of: CD8^+^Ki67^+^ cell infiltrates high (22.8 months) vs. low (7.7 months) (**A**); CD20^+^Ki67^+^ cell infiltrates high (22.8 months) vs. low (12.4 months) (**B**); CD8^+^Tbet^+^ cell infiltrates high (20.5 months) vs. low (10.6 months) (**C**); and RORγt+ cell infiltrates high (18.7 months) vs. low (10.5 months) (**D**). Diminished OS was associated with tumors expressing higher mean intensities of IFNγ (30.9 months) vs. low (14.2 months) (**E**).

We hypothesized that specimens containing increased densities of B and T cells expressing markers of proliferation, differentiation, or function, would associate with favorable prognosis in GBM patients. To assess whether TIL densities and subsets, or markers of immune activity were prognostic for OS, univariate and multivariate Cox proportional hazards analyses were performed. Evaluated immune infiltrates and markers were dichotomized into high and low categories. In univariate analyses, there was no correlation between total densities of infiltrating CD4, CD8, and CD20 and survival (Table 2). However, improved OS was associated with higher densities of proliferating CD8^+^ T cells (CD8^+^Ki67^+^) (HR 0.36, *p* = 0.001) and proliferating B cells (CD20^+^Ki67^+^) (HR 0.51, *p* = 0.008), as well as CD8^+^T-bet^+^ cells (HR 0.46, *p* = 0.004), and total RORγt cells (HR 0.56, *p* = 0.04; Table 2). Conversely, higher mean intensities of IFNγ (HR 2.23, *p* = 0.002), and advanced patient age (HR 1.13, *p* = 0.003) were associated with diminished survival (Table 2). Kaplan–Meier curves for a subset of the analyzed variables are shown in Figure 3A–E.

A multivariable Cox proportional hazards model assessed the relationship between OS and immune cell densities in the context of a set of clinical covariates known to be prognostic that included age, KPS, extent of resection, and MGMT promoter methylation status [2,50,51,52]. In this multivariable analysis, improved OS was associated with high densities of proliferating CD8^+^ T cells (CD8^+^Ki67^+^) (HR 0.15, *p* < 0.001) (Supplemental Figure S6), and a high ratio of CD8^+^ cells to CD4^+^ cells (HR 0.31, *p* = 0.005) (Table 3, and Supplemental Figure S7A,B). Conversely, decreased OS was associated with advanced age (HR 1.21, *p* = 0.005), and higher mean intensities of IFNγ (HR 2.13, *p* = 0.027) (Table 3 and Supplemental Figure S7C,D).

## 4. Discussion

In this study, we found that high densities of proliferating CD8^+^ cells (CD8^+^Ki67^+^) and higher ratios of CD8^+^ TIL relative to CD4^+^ TIL were associated with improved survival by multivariate analysis, in which patient age also had prognostic associations. Our finding that higher ratios of CD8^+^ TIL relative to CD4^+^ TIL were associated with improved overall survival is consistent with prior studies showing enhanced survival associated with high densities of CD8^+^ TIL [53], low densities of CD4^+^ TIL [11], and increased ratios of CD8^+^ TIL to FoxP3^+^ TIL [54]. The balance between CD8 and CD4 TIL populations has prognostic implications in GBM that may be associated with the quality of the antitumor immune response. Interestingly, we also found that high densities of proliferating CD8^+^ cells (CD8^+^Ki67^+^) were associated with improved OS in multivariable analyses, and by univariate analyses that high density of CD8^+^T-bet^+^ cells were prognostic for improved OS. Collectively, these results suggest that a subset of CD8^+^ TIL express markers of T cell activity, which include proliferation and expression of (T-bet), a master transcriptional regulator of effector T-cell activation, and their correlation with improved OS suggests an antineoplastic effect. By univariate analysis, we found that RORγt^+^ cells were associated with improved OS in GBM. While one study has evaluated IL-17A expression in glioblastoma, and found an association between increased IL-17A^+^ infiltrates and diminished OS [55], RORγt expression has not been evaluated in GBM. Thus, studies are needed to delineate whether RORγt^+^ cells can lend a tumor-cytolytic benefit [23,24,25,26].

In the interest of studying a homogenous sample population, in our patient cohort of 77 patients with GBM, we excluded patients that did not receive standard of care therapy, and excluded patients whose tumors had a mutation in IDH1/2, which is associated with prolonged OS [56,57]. A limitation of studies examining the GBM immune environment is that most patients receive pre-operative steroids to control cerebral edema [58,59], and their use has been associated with immune suppression [60]. Although most of the patients included in our study received pre-operative dexamethasone, they were given in short duration (mean of 4.76 days). Consistent with a previous study that suggested that pre-operative corticosteroids might not reduce TIL density in brain metastases [61], we found no significant associations between dexamethasone treatment and CD4^+^, CD8^+^, or CD20^+^ TIL densities. Immunotypes in GBM have not be previously evaluated. We found that infiltrate patterns in GBM were comprised of immunotype A (53%) or B (47%) patterns and no immunotype C tumors were observed. The lack of immunotype C infiltration patterns in GBM highlights the limited infiltrates in GBM and suggest that therapies may be needed that promote diffuse infiltration of T cells.

Although B cells are known to infiltrate GBM, their role remains uncertain. We observed that the density of infiltrating B cells had no prognostic implications, in accordance with a prior study [43]. However, we found that proliferating B cell (CD20^+^Ki67^+^) infiltrates were associated with improved prognosis by univariate analyses, but not by multivariate analysis. TLS are associated with improved survival in several cancers [32], but their prevalence and function in GBM are unknown. One recent study found that TLS were present in 8 of 16 (50%) evaluated GBM, and their presence correlated with an increase in tumor infiltrating T cells [62]. We found evidence of structures resembling immature TLS [47,48,49] in 8% of evaluated tumors. The high frequency of TLS in GBM is in contrast to our study, however. A potential reason for this discrepancy is that the prior study only evaluated GBM that contained meningeal tissue, as TLS were located close to the meninges [62]. Thus, our patient cohort may be more representative of the general population of GBMs. Collectively, these data raise questions about the prevalence of TLS in GBM and function. Additionally, futures studies are warranted to better define TLS in GBM. TLS in other cancers have been identified and defined by expression of a number of chemokines that are involved in TLS neogenesis: CCL19, CCL21, CXCL12, CXCL13, CCL17, and CCL22 [63]. Germinal center identification and maturation in TLS has been defined by markers including: CD21 and CD23 to identify follicular dendritic cells (DCs) and different maturation states of the B cell follicle and activation-induced deaminase (AID), a critical enzyme for somatic hypermutation and class switch recombination of immunoglobulin genes [37,63]. Additionally, TLS often contain mature dendritic cells localized nearby that may aid in antigen presentation [51]. These markers have yet to be assessed in GBM TLS.

IFNγ is an effector cytokine, associated with antitumor mechanisms, which include increased tumor immune surveillance, and antiproliferative, pro-apoptotic tumor mechanisms. Conversely, IFNγ may also play a pro-tumorigenic role, through upregulation of immune suppressing mechanisms including indoleamine 2,3-dioxygenase (IDO), recruitment of Tregs, and upregulation of checkpoint inhibitors, such as programmed cell-death ligand (PD-L1) [22,64,65]. In our study, we paradoxically found that IFNγ intensity in GBM tissue was associated with diminished OS in patients by univariate and multivariable analyses. Additionally, we found that IFNγ was not tightly localized adjacent to CD4^+^ or CD8^+^ T cell infiltrates in GBM, which could indicate that other cells subsets such as NK cells, macrophages, or dendritic cells are producing IFNγ in GBM. Thus, IFNγ may be inducing immune suppressing mechanisms, or it may be associated with other undetermined effects, which may be detrimental, such as inflammatory effects. In line with this, PD-L1 expression has been correlated with IFNγ [66] and worse prognosis in GBM [67]. Thus, blocking immune regulating effects of IFNγ may help to improve prognosis in GBM.

A strength of this study is that we evaluated a large and homogenous cohort of patients, and larger biopsy areas, for T and B cell infiltrates with mIFH, which enables analysis of multiple immune markers together as well as cell distribution and location. However, a limitation of this work is that we visualized fewer immune markers, which were repeated across panels and multiple sections, so that we could comprehensively study B and T cell infiltrates in conjunction with markers of immune activity, differentiation and function. Additionally, we did not include analyses of other cell subsets in GBM with prognostic associations, such as glioma-associated microglia/macrophages (GAMs) [68]. GAMs have been recently identified as the dominate infiltrating immune cell in GBM, and may constitute another immune suppressive mechanism in GBM that needs to be overcome [69]. Thus, studies are need to elucidate the location of GAMs in GBM relative to infiltrating CD8^+^ T cells, and their impact on T cell function.

We have found that the composition and functionality of GBM-infiltrating lymphocytes have prognostic relevance and the potential to inform immunotherapy strategies. A limitation of our study is that further work is needed to elucidate the mechanisms underlying these associations. Additional studies are needed to address whether CD8^+^ T cell proliferation is correlated with later T cell effector functions, such as effector cytokine production in the form of perforin, granzyme-b, or TNF-α, and if not, whether these can be enhanced with immune therapies targeting those functions. Additionally, the dominance of CD8^+^ T cells over CD4^+^ cells in GBM holds prognostic relevance, which needs to be further elucidated, and may be due the presence of CD4^+^ Tregs. Potentially, therapies may be needed to promote diffuse infiltration of proliferating CD8^+^ T cells in GBM. These may include therapies that directly supply T cell chemotactic chemokines, such as CXCL-9, -10, and -11, into tumor or promote their production from tumor cells [70,71,72], or deplete Tregs [73], or therapies that block IFNγ or its induced immunoregulatory effects through blockade of IDO or PD-1.

In summary, the immune profile analysis of a refined cohort of IDH wild type GBM cases revealed that high densities of proliferating CD8^+^ T-cells and high ratio of CD8^+^ to CD4^+^ cells correlated with improved survival. These findings suggest that the that survival may be enhanced by increasing proliferation of tumor-infiltrating CD8^+^ T cells, and that approaches should be designed to promote CD8^+^ T cell dominance in GBM, and potentially mitigate or modify the immunoregulatory effects of IFNγ in the tumor microenvironment. Additionally, strategies that promote infiltration of T cells diffusely into GBM may be necessary, and further characterization of TLS prevalence and function in GBM is needed.

## Figures and Tables

**Table 1:** Summary of Clinical Date.

Clinical Factors	Number (%)
Patients	77
Females (%)	32 (41.6)
Males (%)	45 (58.4)
Age (mean (SD))	61.48 (14.68)
Karnofsky Performance Status	
50	1 (1.3)
60	7 (9.1)
70	33 (42.9)
80	20 (26.0)
90	16 (20.8)
Extent of resection	
Biopsy	7 (9.0)
Subtotal Resection	35 (45.5)
Gross Total Resection	35 (45.5)
Preoperative Dexamethasone Dose (mg/daily)	
0	8 (10.5)
2	1 (1.3)
4	7 (9.2)
6	3 (3.9)
8	5 (6.6)
9	1 (1.3)
12	2 (2.6)
16	13 (17.1)
18	1 (1.3)
24	33 (43.4)
32	1 (1.3)
40	1 (1.3)
	**Mean (SD)**
Preoperative Dexamethasone Dose duration (days)	4.76 (5.34)
Adjuvant Temozolomide cycles	3.80 (3.57)
Bevacizumab cycles	4.96 (9.55)

Abbreviations: SD = Standard Deviation.

**Table 2:** Univariate analysis of evaluated clinical and immunological factors and overall survival.

Factors	Univariate HR	95% CI		*p*-Value
**Sex**	0.78	0.49	1.25	0.3
KPS high	0.85	0.53	1.36	0.5
Age	1.13	1.04	1.22	**0.003**
MGMT Methylation	0.66	0.39	1.13	0.13
Resection extent (vs. biopsy)				
Subtotal Resection	0.95	0.42	2.15	0.9
Gross Total Resection	0.9	0.4	2.03	0.79
DEX dose preoperative	0.99	0.97	1.023	0.84
DEX duration	1	0.96	1.04	0.99
High CD4^+^ cells	1.43	0.85	2.41	0.18
High CD8^+^ cells	1.2	0.75	1.9	0.44
High CD20^+^ cells	0.68	0.41	1.13	0.13
High RORγt^+^ cells	0.56	0.32	0.98	**0.040**
High CD4^+^T-bet^+^ cells	0.8	0.5	1.28	0.35
High CD8^+^T-bet^+^ cells	0.46	0.26	0.79	**0.004**
High CD4^+^Ki67^+^ cells	0.67	0.37	1.2	0.18
High CD8^+^Ki67^+^ cells	0.36	0.2	0.66	**0.001**
High CD20^+^Ki67^+^ cells	0.51	0.31	0.84	**0.008**
High IFNγ^+^ cells	1.51	0.91	2.49	0.11
High ratio of CD8^+^ to CD4^+^ cells	0.66	0.41	1.05	0.08
High mean intensity of IFNγ	2.23	1.33	3.74	**0.002**

The Cox model was used for univariate analysis of immune infiltrates and clinical prognostic factors and associated with OS. Immune infiltrates were enumerated per mm2/tumor and dichotomized into high or low. Clinical factors included KPS (high > 70 vs. low ≤ 70), age (per 5-year increase), MGMT methylation status (yes/no), extent of resection (STR vs. GTR vs. Biopsy), and dexamethasone dose and duration. Hazard ratios (HR) less than 1 reflect longer survival. A *p* value less than 0.05 was considered statistically significant (bold). Definition of abbreviations: DEX = dexamethasone; MGMT = O6-Methylguanine-DNA Methyltransferase; KPS = Karnofsky Performance Status Score.

**Table 3:** Multivariate analysis of evaluated clinical and immunological factors and overall survival.

Factors	Multivariate HR	95% CI		*p*-Value
Age (per 5-year increase)	1.21	1.06	1.38	0.005
Resection extent (vs. biopsy)				
Subtotal resection	0.76	0.27	2.13	0.598
Gross total resection	0.33	0.10	1.05	0.059
KPS high	1.02	0.52	1.98	0.960
MGMT Methylation (yes)	0.74	0.42	1.31	0.310
High CD8^+^Ki67^+^ infiltrates	0.15	0.06	0.38	**<0.001**
High ratio of CD8^+^ to CD4^+^ cells	0.31	0.14	0.71	**0.005**
High mean intensity of IFNγ	2.13	1.09	4.15	**0.027**

The Cox model was used for the multivariate analysis. Immune infiltrates were enumerated per mm2/tumor and dichotomized into high or low. A *p* value less than 0.05 was considered statistically significant (bold). KPS = Karnofsky Performance Status.

## Data Availability

All data are available from the authors on reasonable request.

## Supplementary Materials

The following are available online at https://www.mdpi.com/article/10.3390/cells10123378/s1, Figure S1: Single-plex images of TIL in GBM, Figure S2: Single-plex images of colon and TIL in GBMS1, Figure S3: Single-plex images of colon and GBM stained with TLS panel, Figure S4: Images of regions of interest selection in GBM, Figure S5: Bar Graphs of average tissues sizes analyzed and variance in counts across the mIFH panels, Figure S6: GBM containing high densities of CD8^+^Ki67^+^ cell infiltrates are associated with improved OS, Figure S7: Representative Images of GBM specimens containg low (A) and high (B) ratios of infiltrating CD8^+^ cells relative to CD4^+^ cells, and of GBM specimens containing high (C) and low (D) expression of IFNγ, which correlate with survival, Table S1: Remark Profile of analyzed samples and variables, Table S2: Table of clinical features and TIL infiltrates in patients containing IDH wild-type GBM or GBM with IDH1/2 mutations, Table S3: Dexamethasone treatment and association with CD4^+^, CD8^+^ and CD20^+^ TIL.
