# Supplementary material for: Proliferating CD8+ T Cell Infiltrates Are Associated with Improved Survival in Glioblastoma
Source: Cells. 2021 Dec 1;10(12):3378. doi: 10.3390/cells10123378 (PMC8699921; doi:10.3390/cells10123378)
Supplement: Supplementary file 1 [file cells-10-03378-s001.zip › cells-1451802-supplementary.pdf]

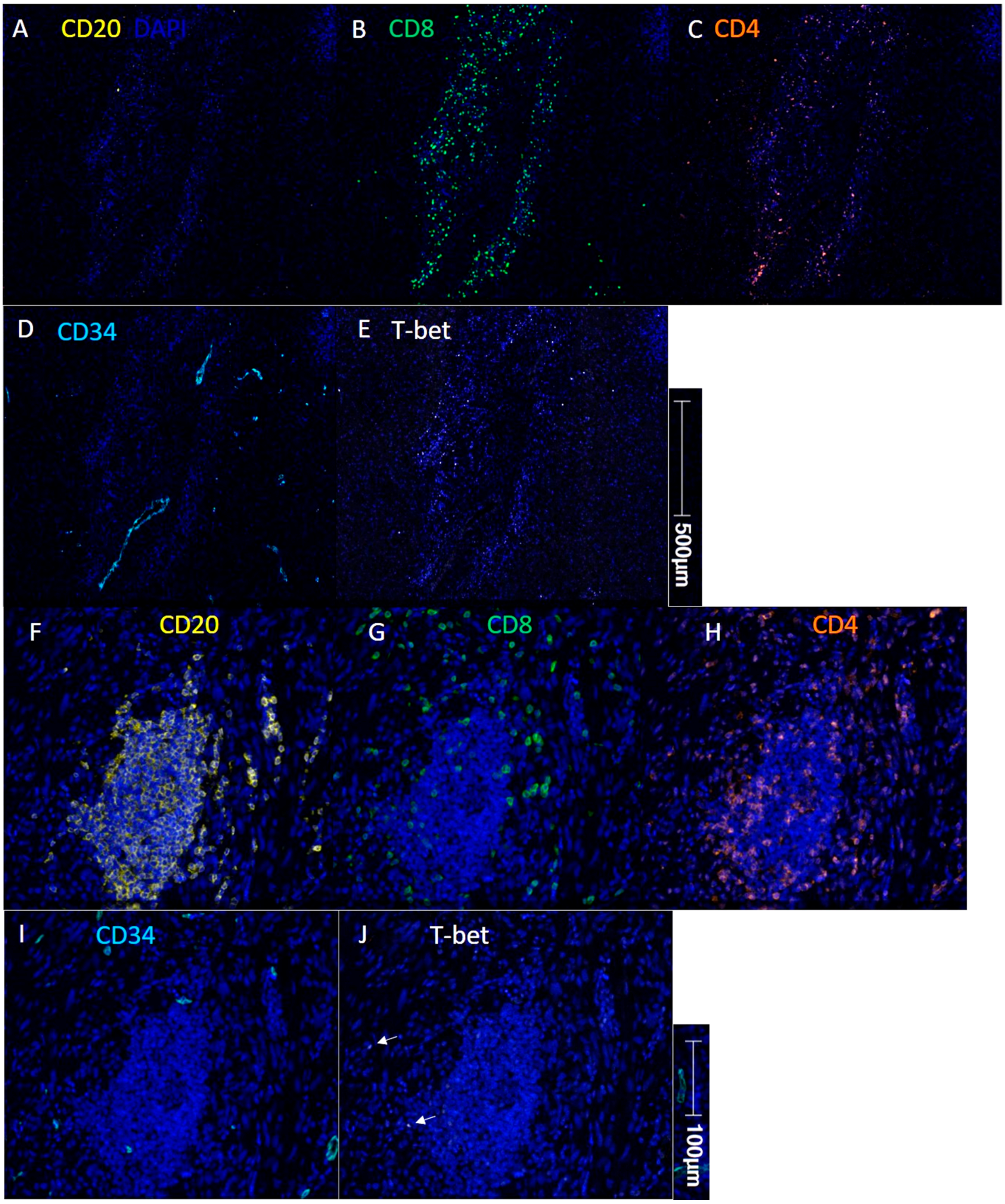

Figure S1. Single-plex images of TIL in GBM. **A–E**) Images are from one representative tumor, and were stained with the TIL panel 1. **F–J**) Images are from a lymph node, utilized as a positive staining control.

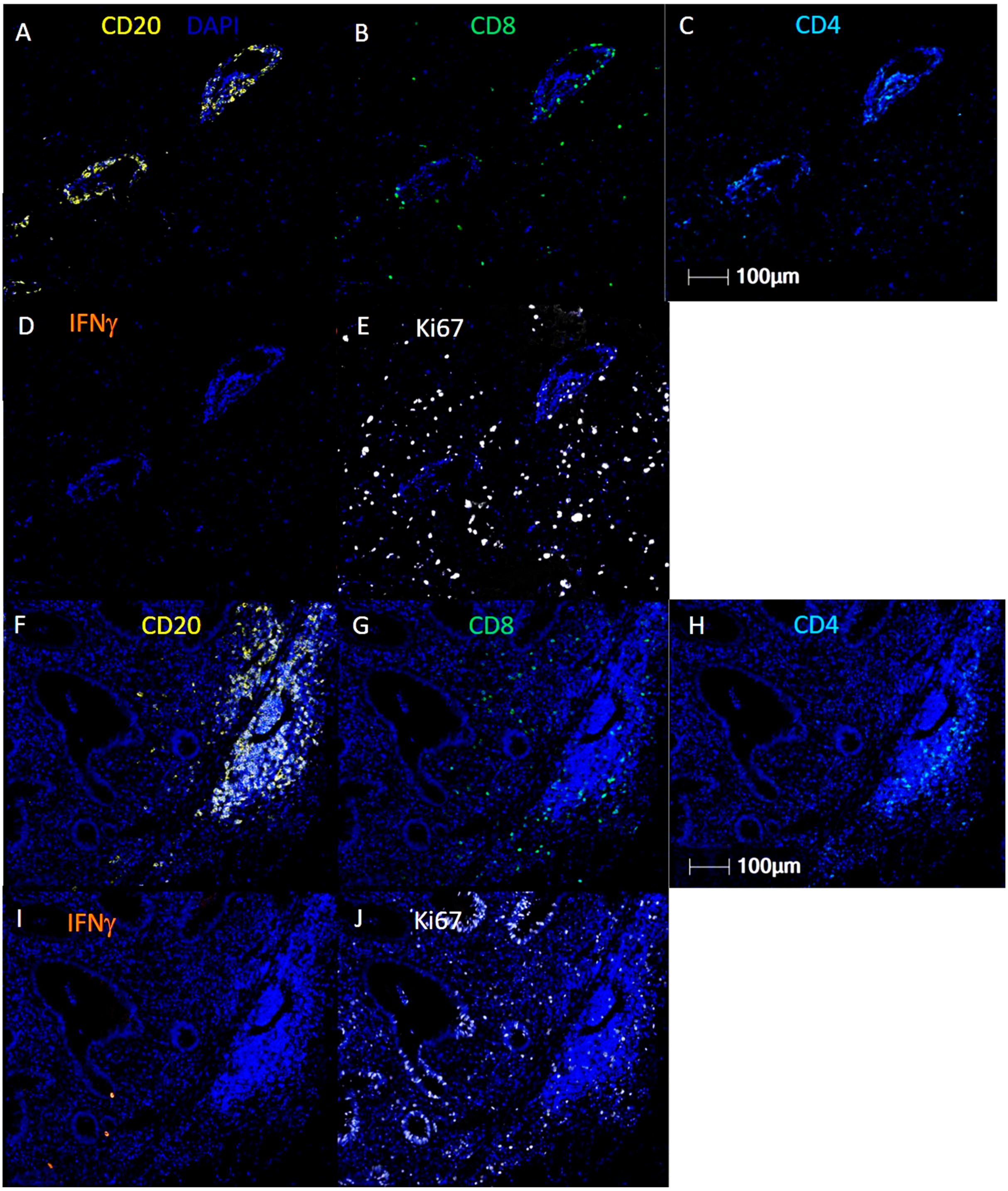

Figure S2. **Single-plex images of colon and TIL in GBM**. **A–E**) Images are from one representative tumor, and were stained with the TIL panel 2. **F–J**) Images are of a Peyer's patch in colon, utilized as a positive staining control.

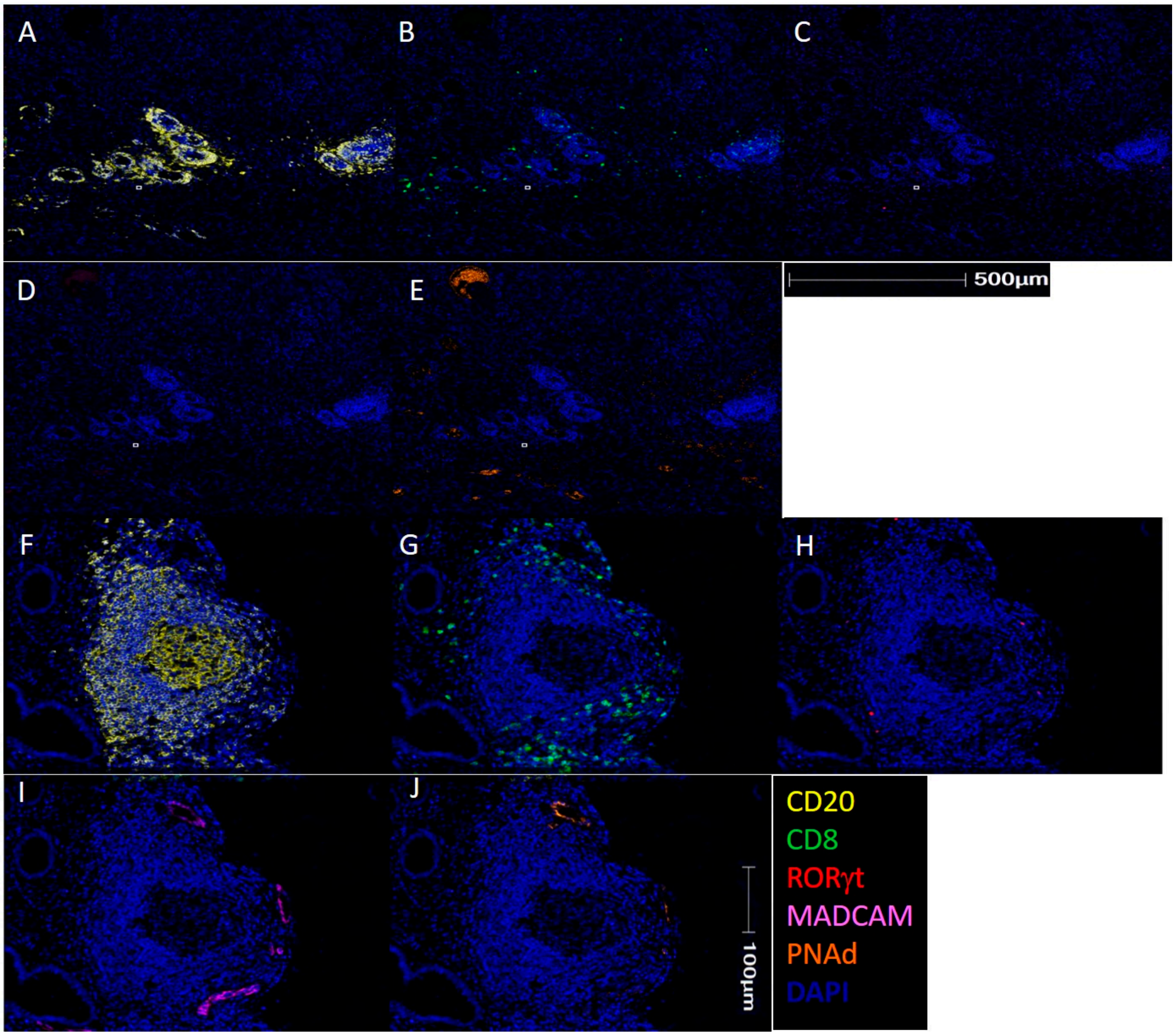

Figure S3. **Single-plex images of colon and GBM stained with TLS panel. A–E**) Images are from one representative tumor. **F–J**) Images are of a Peyer's path in colon, utilized as a positive staining control.

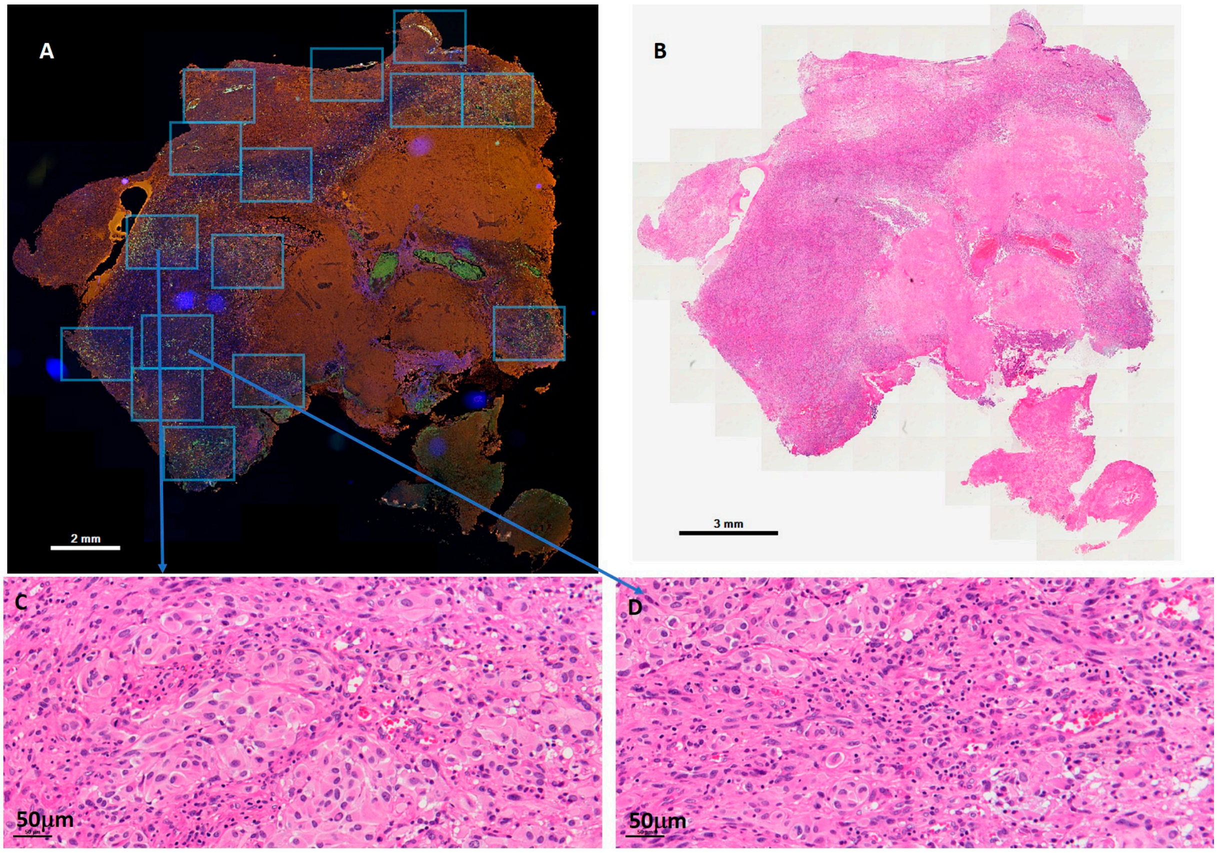

Figure S4. Images of regions of interest selection in GBM. Images are of a multiplex stained tumor **(A)** and H&E-stained tumor **(B–D)**. Regions of interest for analysis, selected on multiplex-stained samples **(A)**, were compared to a serial H&E-stained section **(B–D)** to guide identification of viable tissue regions. Image magnifications are indicated, and **(C–D)** are high magnification H&E images corresponding to the regions of interest selected in **A**.

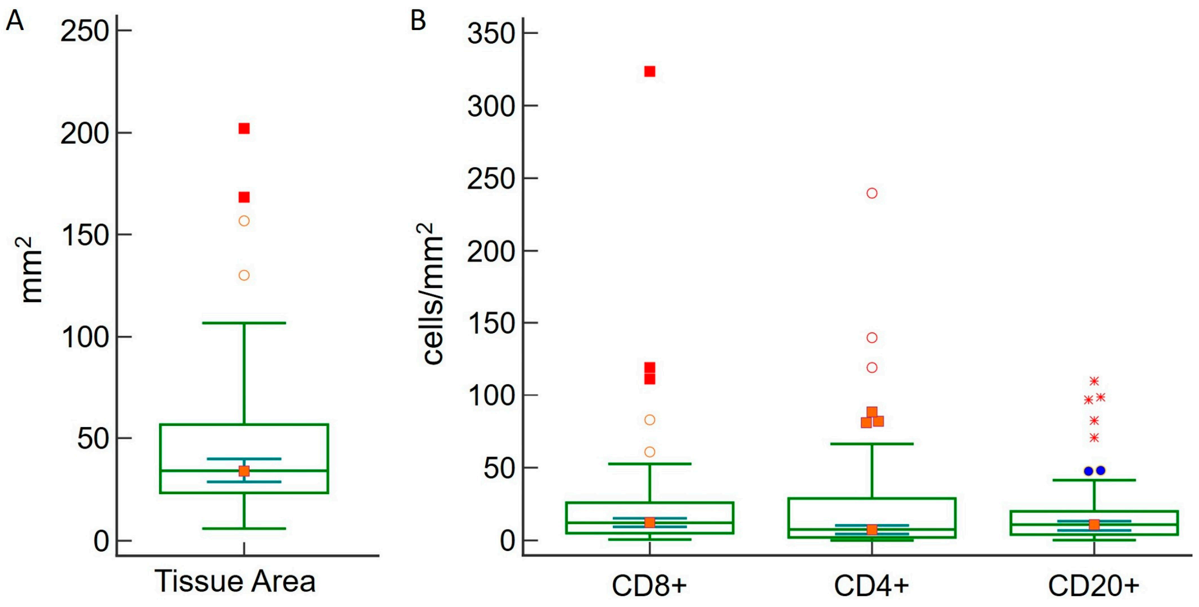

Figure S5. Bar Graphs of average tissues sizes analyzed and variance in counts across the mIFH panels. CD8⁺ cells and CD20⁺ cells were enumerated from 3 staining mIFH panels on 3 serial FFPE sections. CD4⁺ cells were stained in 2 mIFH panels, on 2 serial sections. **A)** Box plot of the average analyzed tissue areas. **B)** Box plot of the variance in cell counts enumerated across the multiple sections. The central box represents values from the lower to upper quartile, 25th to 75th percentile. Middle bar identifies median, and whiskers show minimum and maximum, outliers are displayed as separate points. Spearman's test was used to test correlation in cell counts across panels. We observed significant ($p<0.0001$ for all) and positive correlations between CD4, CD8, and CD20 markers across the panels and serial sections. CD4 cells across 2 mIFH panels ($r=0.78$, $p<0.0001$), CD8 cells across 3 mIFH panels ($r= 0.8$, $r=0.84$, $r=0.85$, $p<0.0001$ for all comparisons, panel 1 to 2, panel 1 to 3, panel 2 to 3), CD20 counts across 3 mIFH panels ($r= 0.89$, $r=0.68$, $r=0.69$, $p<0.0001$ for all comparisons).

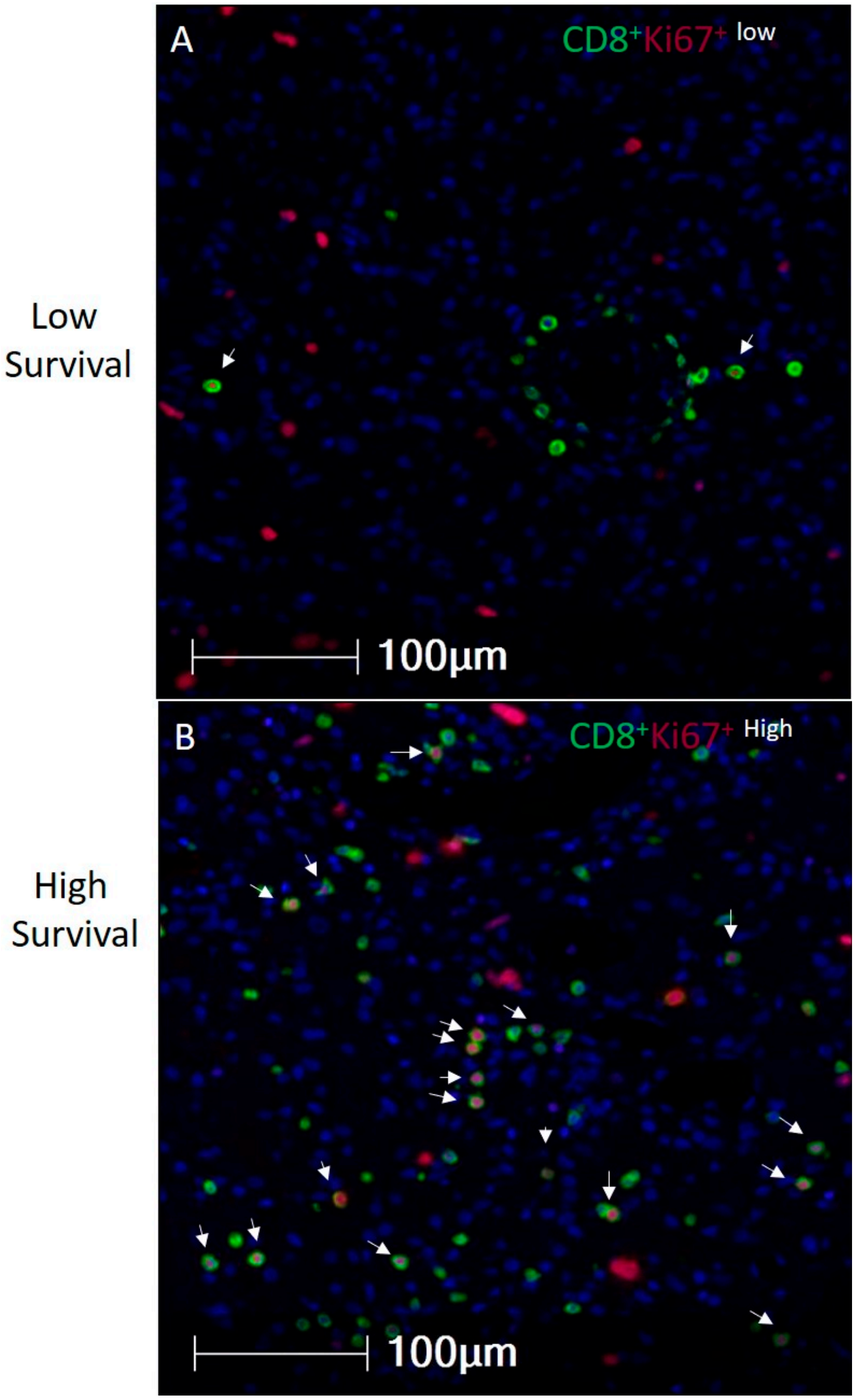

Figure S6. GBM containing high densities of CD8$^+$Ki67$^+$ cell infiltrates are associated with improved OS. Images of GBM specimens containing low (A) and high (B) densities of infiltrating

$CD8^+Ki67^+$ cells, which associate with survival. Dual staining $CD8^+Ki67^+$ cells are denoted by arrows.

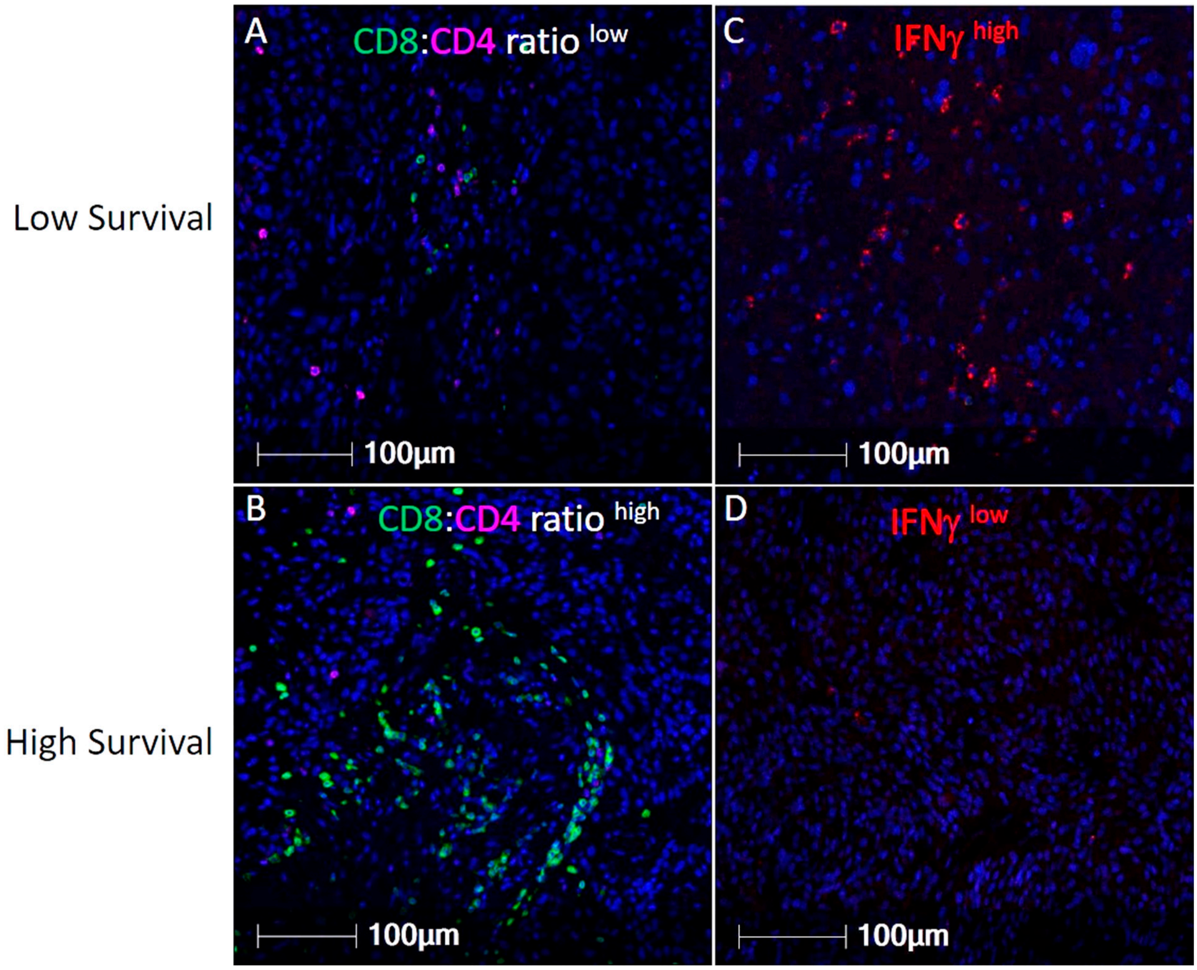

Figure S7. Representative Images of GBM specimens containg low (A) and high (B) ratios of infiltrating $CD8^+$ cells relative to $CD4^+$ cells, and of GBM specimens containing high (C) and low (D) expression of IFNg, which correlate with survival.

Table S1. Remark Profile of analyzed samples and variables

a) Patients treatment and variables

**Study and marker**	**Remarks**
Markers	M1 = CD20^{+}, M2 = CD4^{+}, M3 = CD8^{+}, M4 = ratio of CD4 to CD8, M5 = CD4^{+}Ki67^{+}, M6 = CD8^{+}Ki67^{+}, M7 = CD20^{+}Ki67^{+}, M8 = CD4^{+}T-bet^{+}, M9 = CD8^{+}T-bet^{+}, M10= RORγt^{+}, M11 = IFNγ Intensity
How was marker analyzed?	Cells expressing marker were quantified using Halo digital software (cells/mm^2 tumor) as well as marker intensity.
If categorical, how were cutpoints determined?	Categorical high vs low cut points were determined for each evaluated immunological marker using the Contal O'Quigley method
Further variables	v1 = Age, v2 = MGMT methylation (yes/no), v3= Resection extent Gross Total Resection vs Biopsy, v4= Subtotal Resection vs Biopsy, v5= High KPS Score (> 70), v6= Dexamethasone Dose, v7= Dexamethasone Duration
Outcomes	OS (82 patients)

**Patients**	**n**	**Remarks**
Assessed for eligibility	197	Disease: Glioblastoma
Met Inclusion Criteria	108	Patient Source: University of Virginia Neuro-Oncology tumor bank<br>Sample Source: FFPE specimens available; Met Inclusion Criteria[a]
Excluded	31	General exclusion criteria [b], non-stardard therapy, IDH1/2 mutation
Included	77	
With outcome events	75	

b) Statistical analyses of survival outcomes

**Analysis**	**Patients**	**Events**	**Variables considered**	**Results/remarks**
A1: Univariable	77	75	M1-M13; v1-v7	Table 2<br>OS: v1, M6, M7, M9-M13
A2: Multivariable	77	75	M4, M6, M12 ; v1-v5	Table 3<br>Stepwise selection used to determine final variable in analysis<br>OS: v1, v2, M4, M6

[a] Inclusion criteria: Cases that were diagnosed at least 2 years prior to the study start, and patients that received standard of care treatment for GBM, defined as maximum feasible surgical resection followed by concomitant radiation therapy and temozolomide (TMZ). [b] Exclusion criteria included: specimens were from a second surgery, review of pathology revealed anaplastic oligodendroglioma and not GBM, patient received radiation therapy only or only supportive treatment after surgery, patient did not complete RT and TMZ, and tumor contained IDH1/2 mutation.

Table S2. Table of clinical features and TIL infiltrates in patients containing IDH wild-type GBM or GBM with IDH1/2 mutations.

	IDH1/2 wild-type GBM (n)	IDH1/2 mutant GBM (n)
Patients	77	5
Males	45	1
Females	32	4
MGMT methylated	29	1
MGMT unmethylated	32	4
MGMT Status unknown	16	0
Median Age at diagnosis (years)	64.0	32.0
Median Survival (months)	17.1	48.3

Median TIL densities (cells/mm^2)	IDH1/2 wild-type GBM	stdev	IDH1/2 mutant GBM	stdev
$CD4^+$	6.0	36.4	10.2	9.1
$CD8^+$	26.9	63.7	16.7	11.6
$CD20^+$	10.6	33.0	10.7	13.7
$CD4^+Tbet^+$	0.3	10.2	0.5	0.5
$CD8^+Tbet^+$	0.9	5.3	0.6	0.4
$ROR\gamma t^+$	1.9	6.9	2.7	4.5
$IFN\gamma^+$	1.6	14.8	1.7	3.3
$CD4^+Ki67^+$	1.3	39.8	2.7	2.5
$CD8^+Ki67^+$	4.3	20.2	3.0	1.4
$CD20^+Ki67^+$	0.3	13.9	1.3	0.9
Ratio $CD8^+$ to $CD4^+$	3.0	18.3	2.2	1.5
$IFN\gamma$ Intensity	1.3	0.6	0.8	0.5

stdev, standard deviation

Table S3. Dexamethasone treatment and association with CD4$^+$, CD8$^+$ and CD20$^+$ TIL. Associations between CD4$^+$, CD8$^+$, and CD20$^+$ TIL and Dexamethasone dose, pre-treatment, duration, and total dose (dichotomized into low and high) were assessed using a two-sample T-test. P values of ≤0.05 were considered statistically significant. No significant associations between dexamethasone treatment and CD4$^+$, CD8$^+$, or CD20$^+$ TIL densities were observed.

CD4$^+$ T cells	p-value
Dex dose pre (daily/mg) (mean)	0.29
Dex dose duration days (mean)	0.26
Dex total dose (mg) (mean)	0.55
**CD8$^+$ T cells**	
Dex dose pre (daily/mg) (mean)	0.82
Dex dose duration days (mean)	0.63
Dex total dose (mg) (mean)	0.42
**CD20$^+$ B cells**	
Dex dose pre (daily/mg) (mean)	0.79
Dex dose duration days (mean)	0.6
Dex total dose (mg) (mean)	0.94
